# Pseudoseptic Psoriatic Arthritis With Synovial Leukocytosis > 100,000/μL: A Diagnostic Pitfall Mimicking Iatrogenic Infection

**DOI:** 10.1002/jgf2.70201

**Published:** 2026-11-13

**Authors:** Wahei Uemura, Kazuo Matsui

**Affiliations:** ^1^ General Medicine Center, Shimane University Hospital Izumo Japan; ^2^ Department of Rheumatology Teine Keijinkai Hospital Sapporo Hokkaido Japan

**Keywords:** anti‐CCP antibody, diagnostic errors, pseudoseptic arthritis, psoriatic arthritis, septic arthritis, synovial fluid

## Abstract

A 70‐year‐old woman presented with acute bilateral knee pain and fever following intra‐articular injections. Synovial fluid analysis revealed a white blood cell (WBC) count > 100,000/μL, and initial blood cultures grew coagulase‐negative staphylococci, suggesting iatrogenic septic arthritis. However, synovial cultures remained negative. Re‐evaluation revealed overlooked psoriatic skin lesions and high‐titer anti‐cyclic citrullinated peptide (anti‐CCP) antibodies. The diagnosis was revised to psoriatic arthritis (PsA). This case highlights that PsA can manifest as “pseudoseptic” arthritis with massive synovial leukocytosis. Clinicians should exercise caution in diagnosing septic arthritis based solely on synovial WBC counts, even when the clinical context suggests iatrogenic infection.

## 1 Background

Septic arthritis is a rheumatologic emergency requiring immediate intervention. A synovial fluid white blood cell (WBC) count greater than 50,000/μL and particularly exceeding 100,000/μL is traditionally considered highly specific for bacterial infection [1]. However, relying solely on this cutoff can lead to diagnostic errors, as crystalline arthritis and rarely inflammatory arthritides can mimic this presentation.

We report a case of psoriatic arthritis (PsA) presenting with synovial WBC counts > 100,000/μL and a positive blood culture following intra‐articular injections. This “triple pitfall” (high WBC, positive culture, prior procedure) mimicked iatrogenic septic arthritis, highlighting the critical importance of integrating clinical reasoning with laboratory data.

## 2 Case Presentation

A woman in her 70s presented to the emergency department with difficulty moving due to severe bilateral knee pain. She had a history of maxillary cancer treated 5 years prior. Seven days before admission, she developed knee discomfort, and 6 days prior, she received intra‐articular hyaluronate injections for osteoarthritis at another clinic and was prescribed celecoxib without any antibiotics. Her pain worsened significantly, leading to hospital admission.

On examination, she was alert and hemodynamically stable (blood pressure 130/70 mmHg), but febrile (38.6°C). Both knees were swollen, hot, and tender, with restricted range of motion. Dermatological examination initially focused on the joints, and no specific rash was noted. Laboratory tests revealed elevated C‐reactive protein (33.13 mg/dL) and leukocytosis (11,270/μL). Arthrocentesis of both knees yielded approximately 25 mL of purulent, turbid yellow fluid per joint. Synovial fluid analysis showed a WBC count of 118,000/μL (82% polymorphonuclear leukocytes) in the right knee and 114,400/μL in the left knee. Gram staining of both fluids showed abundant leukocytes (3+) without microorganisms, and no crystals were observed.

Given the fever, synovial WBC > 100,000/μL, and recent injections, iatrogenic septic arthritis was appropriately suspected. Because she was nontoxic with bilateral involvement, emergency surgical debridement was deferred in favor of close monitoring. Blood and synovial fluid cultures were obtained, and intravenous cefazolin (6 g/day) was initiated for suspected septic arthritis. Concurrently, loxoprofen (180 mg/day) was administered for pain control. By Day 3, following arthrocentesis drainage and loxoprofen, her knee pain had decreased by half, enabling knee flexion and walker‐assisted ambulation. On Day 4 (approximately 48 h after laboratory arrival), initial blood cultures turned positive, prompting repeat blood cultures. Final identification on Day 6 revealed discordant isolates across the two sets: methicillin‐susceptible *Staphylococcus warneri* (cefazolin MIC ≤ 4 μg/mL) in one set and methicillin‐resistant *Staphylococcus lugdunensis* (resistant to β‐lactams; vancomycin MIC ≤ 0.5 μg/mL) in the other. In contrast, initial bilateral synovial fluid aerobic cultures (incubated for 3 days) remained negative. On Day 7, although CRP decreased to 8.26 mg/dL, she developed new right wrist arthritis. Repeat bilateral knee arthrocentesis on Day 7 (20 mL right, 10 mL left) again showed negative Gram stains and negative aerobic, anaerobic, and fungal cultures. On Day 8, after two sets of repeat blood cultures (incubated for 7 days) remained negative, cefazolin was discontinued. On Day 9, knee pain had improved to 10%–20% of baseline, although CRP transiently rose to 13.10 mg/dL.

The emergence of asymmetric polyarthritis despite declining knee symptoms prompted diagnostic reconsideration on Day 9. A detailed re‐evaluation revealed neck pain starting 1 month prior, right hand swelling 5 months prior, and a 10‐year history of psoriasis vulgaris treated with topical therapy, with widespread scaly plaques covering > 10% of her body surface area (Figure 1a–c). Furthermore, although the initial synovial fluid on Day 1 was purulent (Figure 2a), knee ultrasonography on Day 10 demonstrated persistent bilateral synovial hypertrophy (Figure 2b). After switching loxoprofen to celecoxib (400 mg/day) on Day 11, she was discharged on Day 16 (CRP 3.58 mg/dL). Further serological testing showed a high titer of anti‐cyclic citrullinated peptide (anti‐CCP) antibody (78.6 U/mL) and weakly positive rheumatoid factor (20 IU/mL). Radiographs showed no erosions. Based on the Classification Criteria for Psoriatic Arthritis (CASPAR), she was diagnosed with PsA. Infliximab was initiated on Day 24, switched at 4 months to upadacitinib (15 mg/day) for refractory skin lesions, and combined at 5 months with bilateral intra‐articular triamcinolone acetonide injections (20 mg/knee) and methotrexate. Over 52 months of follow‐up, she has maintained remission without recurrent infection.

**FIGURE 1:**
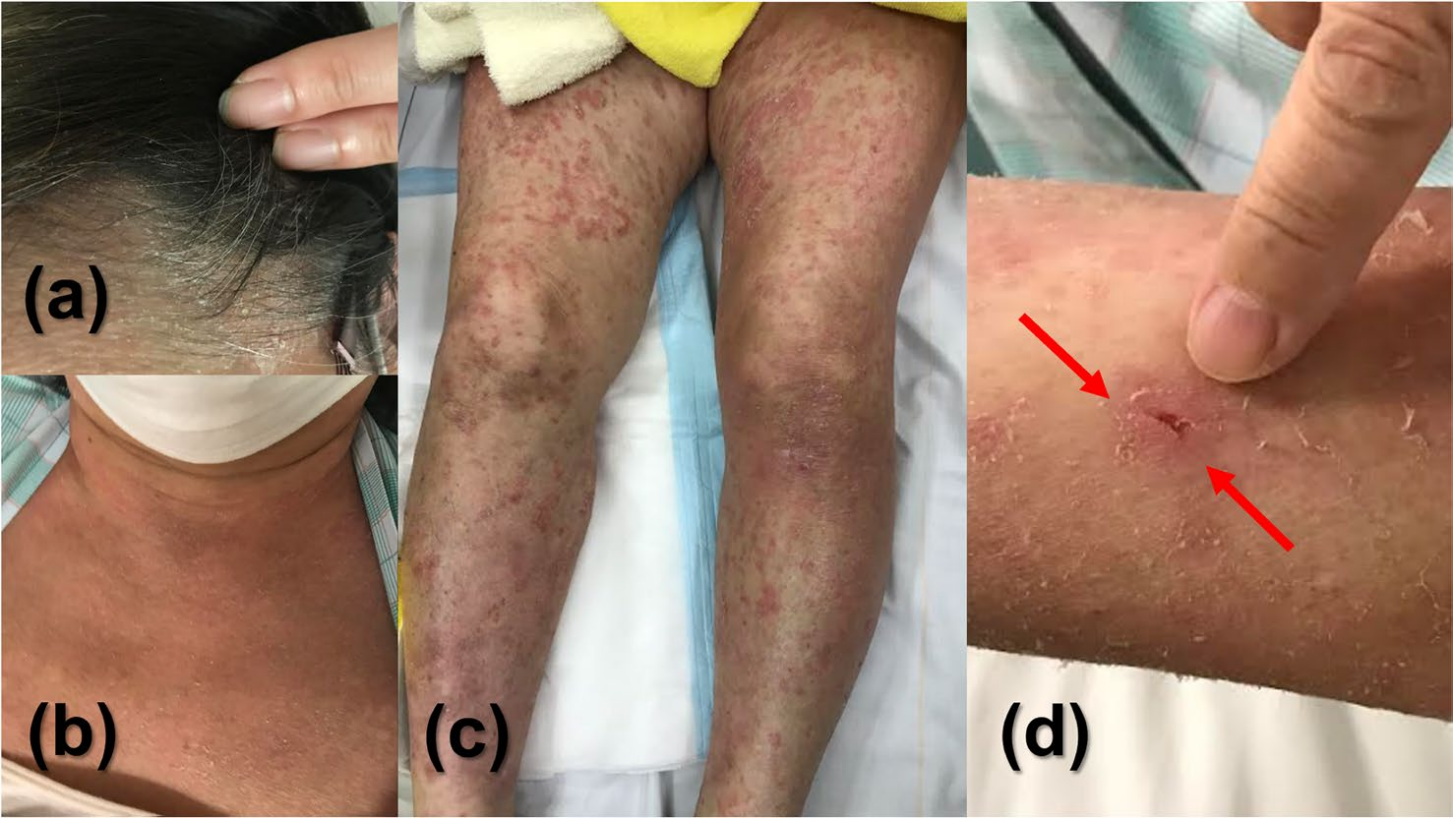
Cutaneous manifestations of psoriatic arthritis. (a) Scaly erythema on the hairline of the scalp. This lesion was initially overlooked due to its hidden location. (b) Erythematous plaques on the anterior chest and neck. (c) Diffuse scaly plaques on the lower extremities. (d) Erosion at the blood culture sampling site on the forearm (red arrows). The development of a psoriatic lesion at the site of trauma suggests the Koebner phenomenon.

**FIGURE 2:**
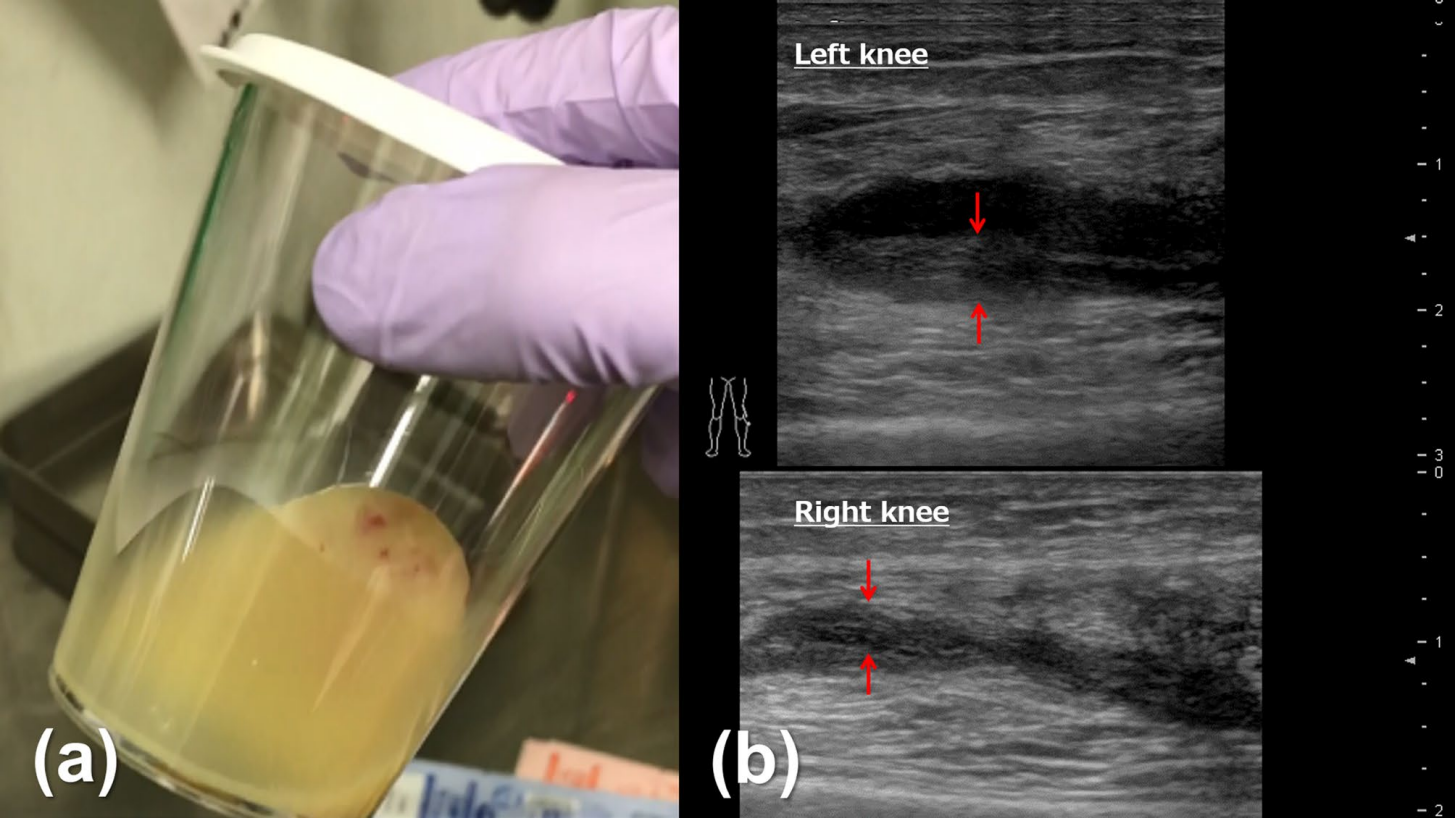
Findings of the knee joints suggesting inflammatory arthritis. (a) Macroscopic appearance of the synovial fluid obtained from the right knee on Day 1. The turbid, yellowish fluid strongly mimicked septic arthritis, leading to the initial administration of antibiotics. (b) Ultrasonography of the knee joints performed on Day 10. Despite antibiotic treatment, significant synovial hypertrophy (red arrows) persisted in both knees, suggesting a non‐infectious, inflammatory etiology.

## 3 Discussion

This case illustrates a rare presentation of PsA mimicking septic arthritis, termed “pseudoseptic” arthritis. Although initiating empirical antibiotics was reasonable and not a diagnostic error, cognitive biases delayed the diagnosis of PsA. Anchoring on the recent injections and synovial WBC > 100,000/μL, combined with confirmation bias from positive blood cultures, led to premature closure—omitting a thorough skin examination under the assumption that dermatological disease was unrelated. Reconsideration was triggered initially by rapid pain improvement with sterile synovial fluid on Days 3–4, and definitively by the emergence of asymmetric polyarthritis on Days 7–9.

The most significant finding is the extreme synovial leukocytosis. A synovial WBC count > 100,000/μL has a positive likelihood ratio of 28.0 for septic arthritis [1], and reports of PsA exceeding this cutoff in immunocompetent older adults are scarce [2]. Importantly, negative cultures alone cannot exclude infection, even without prior antibiotic exposure. Because standard cultures were used without blood culture bottle enrichment, prolonged incubation, or 16S rRNA gene PCR, fastidious microorganisms could not be excluded solely by microbiology.

Although the mechanism of this “pseudoseptic” presentation remains unclear, recent studies suggest specific inflammatory pathways in PsA. Hornum et al. reported that C5a levels are significantly elevated in PsA synovial fluid, driving intense neutrophil migration [3]. Furthermore, Frasca et al. demonstrated that neutrophils in PsA joints release LL37 and form neutrophil extracellular traps (NETs), which can further amplify local inflammation [4]. These mechanisms may explain the massive accumulation of neutrophils mimicking bacterial infection seen in our case.

The positive blood cultures further complicated the clinical picture. Unlike typical coagulase‐negative staphylococci, *S. lugdunensis* exhibits *Staphylococcus aureus* ‐like virulence and can cause true bacteremia even from a single positive set. However, our *S. lugdunensis* isolate was methicillin‐resistant (cefazolin‐insusceptible). True *S. lugdunensis* bacteremia and bilateral septic arthritis could not have resolved without vancomycin or surgical debridement, nor would repeat blood cultures on Day 4 have cleared spontaneously. Rather, initial improvements in pain and CRP were attributable to arthrocentesis drainage and loxoprofen, while venipuncture through desquamative psoriatic plaques on the forearm (Figure 1d) is presumed to have contributed to contamination. Furthermore, 52‐month infection‐free survival under potent immunosuppressants and intra‐articular corticosteroids definitively excluded concomitant septic arthritis.

The third confounding factor, the history of recent intra‐articular injection, warrants discussion as a potential cause of “pseudosepsis.” Intra‐articular hyaluronic acid (IA‐HA) injections can induce Severe Acute Localized Reactions (SALR), clinically indistinguishable from septic arthritis. A 2025 targeted literature review reported the incidence of SALR/pseudosepsis following IA‐HA as 0.48% [5]. Furthermore, the 2021 EULAR recommendations state that the efficacy of IA‐HA for knee osteoarthritis is not fully established, urging caution regarding its risk–benefit profile [6]. In our case, the preceding injection likely acted as a confounding factor, creating a diagnostic overlap between IA‐HA‐induced pseudosepsis and the actual onset of psoriatic arthritis.

Finally, the high‐titer anti‐CCP antibody (78.6 U/mL) is noteworthy. While highly specific for rheumatoid arthritis, anti‐CCP antibodies are present in approximately 13.5% of PsA patients and are significantly associated with a polyarticular and erosive phenotype [7]. Our patient exhibited polyarthritis (knees, wrist, neck), consistent with this high‐risk phenotype.

## 4 Conclusion

While PsA should be considered in acute arthritis with synovial WBC > 100,000/μL, clinicians must also carefully evaluate for concomitant infection. Because neither a diagnosis of PsA nor negative cultures alone suffice to exclude septic arthritis, treatment decisions and antibiotic discontinuation must be guided by thorough physical examination and serial clinical reassessment.

## Author Contributions

**Wahei Uemura:** conceptualization, investigation, writing – original draft, visualization, writing – review and editing, data curation, project administration. **Kazuo Matsui:** conceptualization, investigation, validation, writing – review and editing, data curation, supervision, resources.

## Ethics Statement

Ethics committee approval was not required for this case report in accordance with institutional policies.

## Consent

Written informed consent was obtained from the patient for publication of this case report and accompanying images.

## Conflicts of Interest

The authors declare no conflicts of interest.

## Data Availability

Data sharing not applicable to this article as no datasets were generated or analysed during the current study.
